# Nigrostriatal Degeneration Underpins Sensorimotor Dysfunction in an Inducible Mouse Model of Fragile X-Associated Tremor/Ataxia Syndrome (FXTAS)

**DOI:** 10.3390/ijms26041511

**Published:** 2025-02-11

**Authors:** Emre Kul, Mónica Santos, Oliver Stork

**Affiliations:** 1 Department of Genetics & Molecular Neurobiology, Institute of Biology, Otto-von-Guericke University Magdeburg, 39106 Magdeburg, Germany; emrekul@gmail.com (E.K.); mjpsantos@cnc.uc.pt (M.S.); 2 Center for Behavioral Brain Sciences, 39106 Magdeburg, Germany; 3 Center for Intervention and Research on Adaptive and Maladaptive Brain Circuits Underlying Mental Health (C-I-R-C), Jena-Magdeburg-Halle, 07743 Jena, Germany; 4 German Center for Mental Health (DZPG), Site Jena-Magdeburg-Halle, 07745 Jena, Germany

**Keywords:** FMR1 premutation, neurodegeneration, prepulse inhibition, dopamine, nigrostriatal, substantia nigra, P90CGG mouse model

## Abstract

Fragile X-associated tremor/ataxia syndrome (FXTAS) is a late-onset neurodegenerative disorder caused by moderately expanded CGG trinucleotide repeats in the 5′ untranslated region (UTR) of the *FMR1* gene. Characterized by motor deficits such as action tremor and cerebellar gait ataxia, FXTAS is further distinguished by ubiquitin-positive intranuclear inclusions in neurons and glia. However, its clinical spectrum often overlaps with other neurodegenerative conditions such as Parkinson’s disease (PD). Sensorimotor gating deficits, commonly associated with disorders affecting the nigrostriatal pathway such as PD, have been reported in FXTAS, but the underlying connection between these two phenotypes remains undetermined. In this study, we used the P90CGG mouse model of FXTAS, which expresses 90 CGG repeats upon doxycycline induction, to investigate sensorimotor gating deficits and their relationship to nigrostriatal degeneration. After induction, the P90CGG model exhibited late-onset impairments in prepulse inhibition (PPI), a cross-species measure of sensorimotor gating. These deficits coincided with pronounced nigrostriatal degeneration but occurred without evidence of inclusion formation in the substantia nigra. Our findings highlight nigrostriatal degeneration, which has not previously been reported in animal models of FXTAS, and suggest a potential link to sensorimotor gating dysfunction within the context of the disorder.

## 1. Introduction

CGG trinucleotide repeat expansions in the 5′ untranslated region (UTR) of the fragile X messenger ribonucleoprotein 1 (*FMR1*) gene on the X chromosome is the genetic cause of multiple *FMR1*-linked disorders. Fragile X-associated tremor/ataxia syndrome (FXTAS) is the neurodegenerative member of this monogenic family of disorders and stems from moderately expanded premutation alleles of repeat lengths between 55 and 200 CGGs. FXTAS has an estimated prevalence of 1:4000 in the general population and mainly affects men of age 50 and above, with a penetrance rate of 45%. While X chromosome inactivation reduces the impact of FXTAS in females, female premutation carriers face an increased risk of developing Fragile X-Associated Primary Ovarian Insufficiency (FXPOI) and of passing on a full *FMR1* mutation, which can lead to Fragile X Syndrome (FXS) in their offspring [1,2,3,4,5].

Action tremor and cerebellar gait ataxia make up the major clinical features of FXTAS, and the presence of ubiquitin-positive intranuclear inclusions in neurons and glia are the pathological hallmark of the disorder. However, FXTAS can manifest with a variety of symptoms such as short-term memory problems, executive function deficits, neuropathy, and parkinsonism [4,6]. The likelihood of developing FXTAS rises significantly with age, and the length of the CGG repeats is regarded as a key factor in predicting the onset [7]. While the disorder’s molecular basis is not fully understood, three main pathogenic mechanisms have been proposed. One well-supported mechanism involves RNA toxic gain-of-function, where repeat-expanded *FMR1* mRNA sequesters RNA-binding proteins, leading to their aggregation into intranuclear inclusions and to their functional depletion, which disrupts cellular homeostasis. A second mechanism is repeat-associated non-AUG (RAN) translation of *FMR1* premutation mRNA, producing homopolyglycine (FMRpolyG) that forms initially cytoplasmic aggregates and contributes to FXTAS intranuclear inclusions. The third, less-studied mechanism involves the formation of R-loops harboring RNA/DNA hybrids that can lead to local DNA damage and cause genomic instability during replication [4,8,9,10].

Sensorimotor gating is the brain’s ability to filter unnecessary sensory stimuli by inhibiting responses to irrelevant or repetitive inputs, enabling focus on important information. Impairments in sensorimotor gating are often associated with disorders that affect the nigrostriatal pathway and midbrain dopamine function, such as Parkinson’s disease (PD). Many classic features of PD, including parkinsonism and nigrostriatal degeneration can manifest in FXTAS, which has led to misdiagnosis in the past [11,12,13,14]. Similarly, sensorimotor gating deficits have been reported in FXTAS patients, with severities correlating with both CGG repeat size and the overall progression of FXTAS [15].

In this study, we sought to describe the sensorimotor gating phenotype and nigrostriatal degeneration in the well-documented inducible mouse model of FXTAS that expresses a premutation-size repeat tract of 90 CGGs in the brain following doxycycline (dox) consumption. This model, hereafter referred to as P90CGG, captures the inclusion pathology of FXTAS in several brain regions and the motor phenotype upon 12 weeks of dox induction [16,17] and has been successfully used in the past as a testing platform for treatment strategies for FXTAS [18,19]. Using the P90CGG model, we identified late-onset deficits in prepulse inhibition (PPI), a cross-species measure of sensorimotor gating, which are accompanied by nigrostriatal degeneration. To align our findings with the existing literature in FXTAS and Parkinson’s disease, we began by evaluating dopamine transporter (DAT) levels in the entire striatum and subsequently focused on dopaminergic neurons in the substantia nigra. Strikingly, the neuronal degeneration in this system occurred in the absence of inclusion formation in the substantia nigra.

## 2. Results

**Figure 1:**
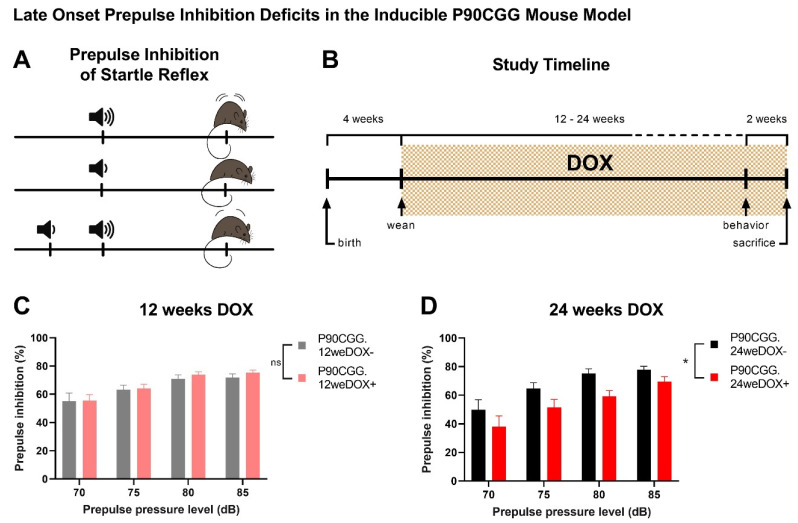
Late-onset prepulse inhibition deficits in the inducible P90CGG mouse model. (**A**) Illustration depicting prepulse inhibition of the acoustic startle reflex in mice, where a mild, non-startling acoustic pulse preceding a strong pulse reduces the startle response triggered by the stronger pulse. (**B**) Timeline showcasing the dox-induction schedule used in the study. Two different schemes have been used with the dox-induction period lasting either 12 weeks or 24 weeks. (**C**) Prepulse inhibition levels in P90CGG mice induced with doxycycline for 12 weeks were unaffected compared to non-induced mice. (**D**) Prepulse inhibition levels were significantly reduced in the dox-induced mice for 24 weeks as compared to their non-induced counterparts. Data presented as mean ±S.E.M. ns: *p* > 0.05, * *p* < 0.05.

Sensorimotor gating problems have been reported both in FXTAS patients and animal models of the disorder [15,20]. Prepulse inhibition (PPI) as a measure of sensorimotor gating is typically observed in an acoustic setting in rodents by recording changes to the startle reflex elicited by acoustic pulses [21,22]. Prepulse inhibition of the acoustic startle reflex can be observed when a mild, non-startling acoustic pulse preceding a strong acoustic pulse triggers a lower startle response than the stronger pulse alone (Figure 1A).

### 2.1. Dox Induction for 24 Weeks Results in PPI Deficits in P90CCG Mice

To investigate whether sensorimotor gating deficiencies observed in FXTAS are modelled in the inducible P90CGG mouse model, we induced the expression of the 90CGG repeat tract in the brain by supplying doxycycline (dox) in drinking water, either for a period of 12 weeks or for 24 weeks, starting at four weeks of age (Figure 1B). When weaker prepulses of 70, 75, 80, and 85 dB were played in an acoustic startle setup preceding the 110 dB main pulse, no differences were observed in PPI levels between 12-weeks dox-induced P90CGG mice and their non-induced littermates (Figure 1C; N(dox−) = 11, N(dox+) = 11, two-way RM ANOVA, dox-effect; F(1,20) = 0.2725, *p* = 0.6074). However, 24 week-dox-induction mice displayed significantly lower PPI in response to the same experimental conditions compared to the dox− control mice (Figure 1D; N(dox−) = 11, N(dox+) = 13, two-way RM ANOVA, dox-effect; F(1,22) = 4.992, *p* = 0.0359). To exclude the potential effects related to dox consumption in general and to confirm the repeat-length dependency of the PPI phenotype, we subjected P11CGG mice that upon dox induction, express a CGG-repeat tract of eleven repeats to the same experimental setting under the 24-week induction period. The PPI levels of the P11CGG mice dox-induced for 24 weeks were not significantly different from the dox− control group (Supplementary Figure S1A; N(dox−) = 7, N(dox+) = 9, two-way RM ANOVA, dox-effect; F(1,14) = 4.054, *p* = 0.0637). Furthermore, there were no differences in the basal startle responses between dox-induced and non-induced mice across any of the tested groups: P90CGG mice on the 12-week dox schedule (Supplementary Figure S1B, two-way RM ANOVA, dox-effect; F(1,20) = 0.001249, *p* = 0.9722), P90CGG mice on the 24-week schedule (Supplementary Figure S1C, dox-effect; F(1,22) = 0.2033, *p* = 0.6565), or P11CGG mice on the 24-week schedule (Supplementary Figure S1D, dox-effect; F(1,14) = 0.5299, *p* = 0.4786). Moreover, basal startle responses increased in all groups with increasing sound pressure level (SPL) of the presented pulses, indicating intact hearing (P90CGG 12-week group: two-way RM ANOVA, SPL-effect; F(1.436,28.71) = 29.71, *p* < 0.0001; P90CGG 24-week group: SPL-effect; F(1.195,26.30) = 40.33, *p* < 0.0001; P11CGG 24-week group: SPL-effect, F(1.556,21.78) = 29.76, *p* < 0.0001).

### 2.2. Nigrostriatal Involvement Accompanies PPI Deficits in P90CGG Mice

**Figure 2:**
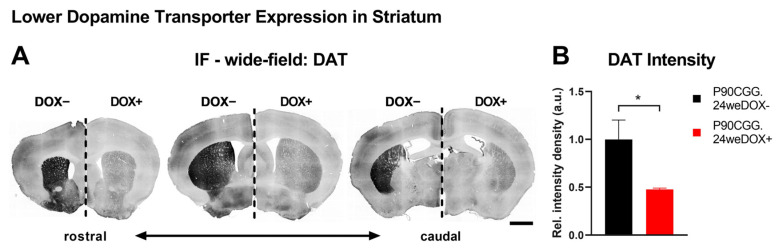
Lower dopamine transporter expression in striatum. (**A**). Representative photomicrograph of coronal brain sections across rostrocaudal axis from dopamine transporter (DAT) immunofluorescence experiments showing contrasting striatal DAT signal (black) in the 24-week-dox-induction versus non-induced P90CGG mice. For each representation, the left side is the dox− control section, and the right side is a section from a dox-induced mouse. Scale bar indicates 1 mm. (**B**). DAT signal quantified from the entire striatum was significantly reduced in the sections obtained from 24-week-dox-induction P90CGG mice as compared to their non-induced counterparts. Data presented as mean ±S.E.M. * *p* < 0.05.

**Figure 3:**
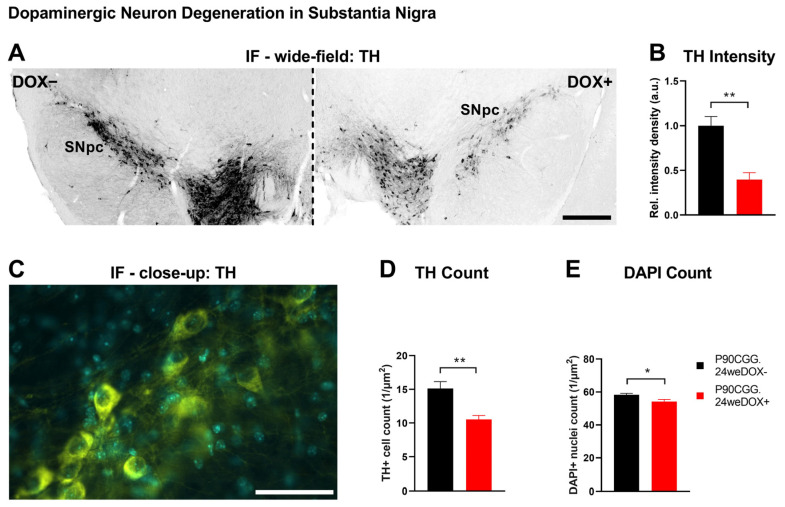
Dopaminergic neuron degeneration in substantia nigra. (**A**). Representative photomicrograph of coronal brain sections from tyrosine hydroxylase (TH) immunofluorescence experiments showing altered TH signal (black) in the substantia nigra pars compacta (SNpc) of the 24-week-dox-induction and non-induced P90CGG mice. Left: dox− control section, right: section from dox induced mouse. Scale bar indicates 500 µm. (**B).** TH signal quantified from the SNpc was significantly reduced in the sections obtained from 24-week-dox-induction P90CGG mice as compared to non-induced control mice. Data presented as mean ±S.E.M. ** *p* < 0.01. (**C**). High-magnification representative photomicrograph from TH immunofluorescence experiments used for cell count quantifications in the SNpc. Yellow: TH, blue: DAPI. Scale bar indicates 50 µm. (**D**). Number of TH immunoreactive cells in the SNpc of the 24-week-dox-induction P90CGG mice was significantly reduced as compared to non-induced control mice. (**E**). Overall number of cell nuclei (DAPI signal) in the SNpc of the 24-week-dox-induction mice was significantly lower compared to non-induced control mice. Data presented as mean ±S.E.M. * *p* < 0.05, ** *p* < 0.01.

Reduced density of striatal dopamine transporter (DAT) is a molecular characteristic linked to nigrostriatal dysfunction, which is observed with varying degrees of severity in patients with FXTAS [23,24] and Parkinson’s Disease (PD), and serves as an alternative diagnostic criterion for PD [25,26,27,28]. This decrease has been associated with PPI deficits in PD patients [29] and in DAT-deficient and knock-out mouse models [30,31]. Moreover, among the various dopaminergic pathways in the brain, dopamine deficiency in the nigrostriatal pathway is specifically linked to reductions in PPI [32]. Thus, we focused our investigation on the nigrostriatal pathway and first evaluated DAT expression levels in the striatum of the PPI-deficient 24-week-induction P90CGG mice via immunofluorescence (Figure 2A). We quantified the fluorescence signal associated with DAT immunoreactivity in the striatum from coronal serial sections. Significantly lower signal intensity density in the striatum was observed with dox-induced mice as compared to non-induced control mice (Figure 2B; N(dox−) = 4, N(dox+) = 4, Mann–Whitney U-test, U = 0, *p* = 0.0286). Since DAT in striatum is presynaptic and contributed by the dopaminergic neurons of the nigrostriatal pathway residing in substantia nigra pars compacta (SNpc) [27], we next examined SNpc (Figure 3A). We quantified the fluorescence signal associated with tyrosine hydroxylase (TH) immunoreactivity, which is the key enzyme for the biosynthesis of dopamine and a widely used molecular marker of dopaminergic neurons in SNpc. The TH signal intensity density was significantly reduced in the SNpc of the dox-induced mice compared to non-induced control mice (Figure 3B; N(dox−) = 4, N(dox+) = 4, Student’s *t*-test, t = 4.667, df = 6, *p* = 0.0034). Although reduced TH expression levels alone may cause dopamine deficiency and lead to neuronal dysfunction [33], we conducted additional analysis by counting TH-immunoreactive neurons in the SNpc to determine whether low TH levels are linked to neuronal loss in our model (Figure 3C; N(dox−) = 4, N(dox+) = 4). Indeed, we observed significantly lower numbers of TH+ neurons in the SNpc of the dox-induced mice as compared to the dox− controls (Figure 3D; Student’s *t*-test, t = 3.904, df = 6, *p* = 0.0080). On the other hand, we also quantified total cell nuclei in the same region via DAPI staining, given that TH immunoreactivity was already reduced in dox-induced mice. The total number of nuclei in the SNpc was also significantly lower for dox-induced mice than for non-induced controls (Figure 3D; Student’s *t*-test, t = 2.687, df = 6, *p* = 0.0362).

### 2.3. Nigrostriatal Degeneration Occurs in the Absence of Inclusions in TH+ Neurons

**Figure 4:**
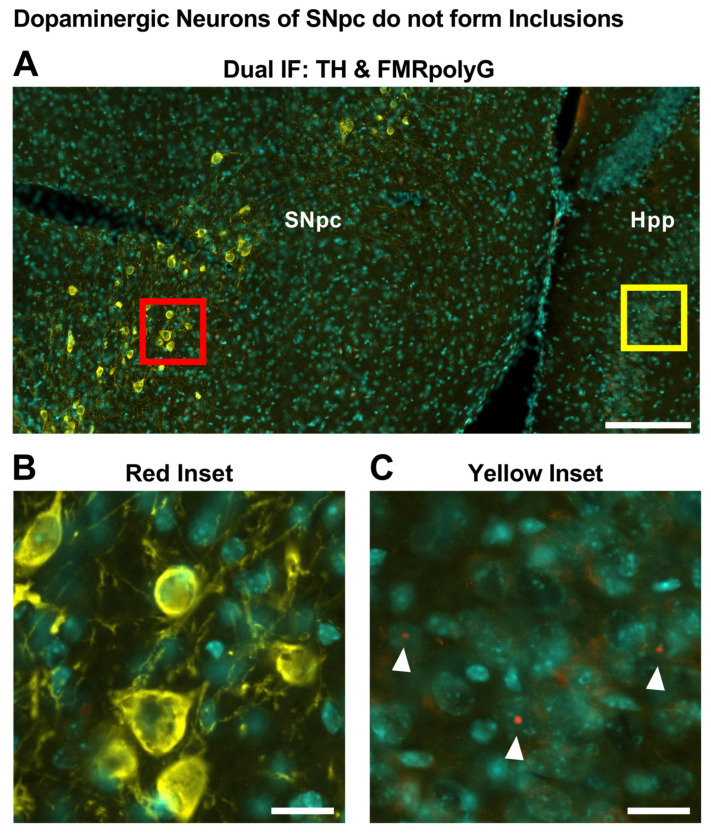
Dopaminergic neurons of SNpc do not form inclusions. (**A**). Representative photomicrograph from dual immunolabelling experiments of coronal sections capturing substantia nigra and parts of hippocampus of 24-week-dox-induction P90CGG mice. Yellow: TH, red: FMRpolyG, blue: DAPI. Scale bar indicates 200 µm. (**B**). High-magnification capture from A (red inset), indicating lack of FMRpolyG foci in the TH immunoreactive cells in SNpc. Scale bar indicates 20 µm. (**C**). High-magnification capture from A (yellow inset), showing FMRpolyG immunoreactive intranuclear inclusions in the hippocampus (Hpp). White arrowheads: FMRpolyG foci. Scale bar indicates 20 µm.

Since intranuclear inclusions in neurons and glia are a hallmark of FXTAS and they are considered to represent a toxic mechanism [10], we next attempted to quantify the number of inclusions in the dopaminergic neurons of SNpc via dual immunofluorescence for TH and FMRpolyG (Figure 4A). Unexpectedly, we observed that the SNpc lacked FMRpolyG+ intranuclear inclusions entirely, including within TH+ dopaminergic neurons. To rule out technical limitations that may lead to inaccurate detection of inclusions, we expanded our analysis to include brain sections containing both the SNpc and the hippocampus, an area previously reported to develop intranuclear inclusions in the P90CGG model [16]. In brain sections of dox-induced mice containing both the SNpc and hippocampus, the TH signal in the SNpc did not colocalize with the FMRpolyG signal (Figure 4B); however, FMRpolyG+ inclusions were detected in the hippocampus (Figure 4C). Additionally, we confirmed that the expanded CGG-repeat tract is expressed in the TH+ neurons following dox-induction via dual immunofluorescence labelling of TH and the GFP reporter [16] in the SNpc of P90CGG mice (Supplementary Figure S2A–D).

## 3. Discussion

FXTAS is a severe neurodegenerative disorder for which no cure currently exists. Thus, preclinical studies are crucial for elucidating the pathology and progression of the disorder. Such research could provide a foundation for developing effective treatment strategies and assessing their therapeutic potential. P90CGG mice represent an inducible model of FXTAS that faithfully recapitulates multiple features of the disorder including the pathology, behavioral phenotype, and progressivity [16,17]. This model has been extensively studied and described by multiple studies and served as a testing platform for several treatment strategies [18,19]. Despite these efforts, sensorimotor gating deficits in the P90CGG model have not been previously described.

Deficits in prepulse inhibition (PPI) alongside reduced striatal dopamine transporter (DAT) levels are well-documented in patients with Parkinson’s disease (PD) [29]. Although similar PPI impairments have been observed in FXTAS patients [15], a connection to the nigrostriatal pathway or the dopamine signalling in general has yet to be established. Additionally, PPI deficits have been reported in the 118CGG knock-in (KI) model of FXTAS and the *Fmr-1* knock-out (KO) model of FXS [20]. In these cases, the phenotype was largely attributed to the reduced FMRP levels in KI mice and the absence of FMRP in the KO model. Notably, in the KO model, PPI deficits emerge early, while in KI mice they manifest later under prolonged premutation expression, a progression that aligns closely with our findings in P90CGG mice. However, it is important to consider that the P90CGG transgene expression occurs out of the context of the *Fmr-1* gene [16].

We for the first time report nigrostriatal degeneration in a mouse model of FXTAS and propose a connection to the sensorimotor phenotype based on the strong association of these features in related neurodegenerative disorders. However, the pathways underlying PPI involve multiple brain regions [21], and we thus cannot exclude contributions from pathological processes occurring in cell types beyond dopaminergic neurons in the substantia nigra pars compacta (SNpc). In fact, the images used for signal intensity measurements indicate that there may be reductions in DAT covering both the dorsal and the ventral striatum (Figure 2) and of TH extending into the ventral tegmental area (Figure 3). Additional studies are warranted to evaluate further cell types that participate in dopaminergic pathways, such as striatal medium spiny neurons, in relation to progressivity of FXTAS in the P90CGG mouse model.

The cerebellum’s role in modulating motor responses and fine-tuning reflexes suggests its potential involvement in PPI deficits, particularly in the P90CGG model. This model exhibits cerebellar pathology as early as 8 weeks after dox induction, with motor deficits emerging by 12 weeks. The cerebellum’s involvement in the manifestation of the acoustic startle reflex (ASR) has been documented in rodents [34,35], raising the possibility that cerebellar dysfunction could indirectly influence PPI through ASR. However, these studies suggest that the cerebellum is primarily involved in processes like habituation rather than the initial response to acoustic stimuli. Consistent with this, we observed no differences in ASR amplitudes between dox-induced and uninduced P90CGG mice (Supplementary Figure S1), suggesting that the cerebellum is not the primary factor underlying the observed PPI deficits.

The fluorescence signal intensity quantifications we performed in the striatum and SNpc revealed stark contrasts between P90CGG dox-induced and control mice. Moreover, the reduction in TH+ cell counts observed in dox-induced mice indicates a degenerative process extending toward cell death. However, as TH expression levels in the SNpc of dox-induced mice were already markedly low, it is possible that our cell count analysis may have overlooked a subset of neurons with minimal TH expression. This is supported by the differences observed between TH+ cell counts (~30% reduction) and overall cell nuclei counts (~7% reduction) in SNpc. Conversely, a reduction in the TH+ neuronal population within the SNpc is likely to be underestimated when quantifying the overall cell population.

It has previously been reported that the development of inclusions is widely seen across cell types in FXTAS patients or in mouse models [36,37]. In fact, neuronal loss and intranuclear inclusions in the neurons of the substantia nigra have been reported in FXTAS patients [11,36]. However, despite the 90CGG transgene expression and its apparent effect on the TH+ cell population, we have not observed any FMRpolyG+ inclusions in residual cells of the SNpc. Strikingly, this seems to be cell-type specific as aggregates at did form the same time in other regions of the transgenic mouse brains.

The proposed mechanisms driving formation of FXTAS inclusions are shown to be toxic to cells in numerous models [38,39,40,41,42]. However, the severity of this toxicity may be influenced by cell type-specific dynamics associated with the expression of expanded CGG repeats. Moreover, the accumulation of toxic proteins or polypeptide fragments within nuclear inclusions may decrease the pool of harmful moieties in a diffuse state within the cell, thereby offering a protective effect [43,44]. In addition to expanded repeat RNA, FXTAS inclusions show enrichment of proteins involved in RNA binding, protein turnover, and DNA damage repair over total nuclear protein composition [45], suggesting that inclusion formation may be a selective, non-incidental process. The intriguing question therefore arises as to whether the presence of inclusions may confer a protective effect. The presence of FMRpolyG within intranuclear inclusions further substantiates this concept, as RAN translation products must undergo active nuclear translocation to become sequestered within the inclusions.

From a translational standpoint, it is important to note that the P90CGG is an artificial model that following dox-induction, overexpresses an expanded CGG-repeat tract independently of the native *Fmr1* gene [16]. As a result, the pathology surrounding the dopaminergic neurons of the nigrostriatal pathway may not accurately reflect the human condition. Additionally, our study does not differentiate the individual impacts of prolonged CGG-repeat expression and normal ageing on the observed PPI deficits, leaving potential interactions between the FXTAS genetic profile and the natural aging processes unestablished. Given the progressive nature of FXTAS, as previously documented in the P90CGG model [16,17,19], it is possible that mild nigrostriatal degeneration already occurs after 12 weeks of dox-induction but remain too subtle to produce detectable sensorimotor gating abnormalities. This parallels earlier findings on the cerebellar phenotype, where shorter dox-induction periods led to pathology without evident behavioral deficits [17]. Consequently, it remains an open question whether the nigrostriatal phenotype can be rescued by earlier intervention, such as transgene shutdown after 12 weeks of dox exposure. Nevertheless, the pronounced nigrostriatal phenotype following 24 weeks of dox-induction is a promising finding, suggesting a novel target for future preclinical studies on therapeutics and rescue strategies using the P90CGG model.

## 4. Materials and Methods

### 4.1. Animals

All mice were bred and kept at the Institute of Biology, Otto-von-Guericke University Magdeburg, Germany, under standard laboratory conditions with a reversed 12 h dark/light cycle. Four weeks after birth, mice were weaned, randomly assigned to experimental and control groups in either the 24-week or the 12-week DOX-induction schedules, and further group housed, ensuring each litter was represented in both experimental groups. Only male mice were used in the study. Group sizes were calculated via the resource equation approach. All animals generated for the study were included in the testing, and no subjects were excluded from the results. Mice were given unlimited access to food (R/M-H V-1534, Ssniff, Soest, Germany) and water, either containing 5% *v/v* sucrose (Roth, Karlsruhe, Germany) or containing 5% sucrose and 4 mg/mL doxycycline hyclate (Sigma, Taufkirchen, Germany). Tail biopsies were taken for genotyping, and earmarks were introduced for individual identification. Genotyping was conducted following the method described by Hukema and colleagues [16]. All procedures were approved by the local ethics committee Landesverwaltungsamt Sachsen-Anhalt (07.10.2013, CEEA# 42502-2-1219UniMD) and complied with both local and European guidelines (EU directive no. 2010/63/EU). Animal experiments were conducted during the active phase of the mice.

### 4.2. Acoustic Startle Response and Prepulse Inhibition

Mice were placed in a holder on a motion sensor inside a padded soundproof chamber. Startle responses were measured and recorded using SOF-825 startle reflex system (Med Associates, Fairfax, VT, USA). Both ASR and PPI measurements were taken on the same day in a protocol lasting 35 min, with inter-trial intervals randomized between 10–30 s and a constant 62 dB white noise background. The experiment began with a five-minute acclimation, followed by 50 ASR trials, where 20 ms white noise pulses (1 ms rise time) at 0, 80, 90, 100, and 110 dB were presented in pseudo-random order, with 10 repetitions per level. Startle responses were then recorded over 500 ms. In the PPI trials, 4 ms white noise prepulses (1 ms rise time) at 0, 70, 75, 80, and 85 dB were followed by 20 ms 110 dB pulses, with a constant 100 ms inter-pulse interval. Each prepulse level was tested in 10 trials, and data were collected for 450 ms, following each 110 dB pulse. Startle responses were quantified by peak amplitudes recorded by SOF-825, with ASR results reported as mean relative amplitudes of 10 trials per pulse level normalized to the mean amplitude of ten 0 dB trials. PPI results were calculated as the average percentage PPI in ten trials per prepulse level using the formula: [(response to pulse alone—response to prepulse plus pulse)/response to pulse alone] × 100. Between tests, holders were cleaned with soapy water. The procedure was adapted from Valsamis and Schmid [22].

### 4.3. Tissue Processing and Immunofluorescence

Mice were anesthetized with a ketamine (80 mg/mL) and xylazine (6 mg/mL) mixture (Sigma) at 1 mg/kg body weight and perfused transcardially, first with phosphate buffered saline (PBS) and then with 4% (*m*/*v*) paraformaldehyde (PFA) in PBS. The brains were extracted, placed in 4% PFA/PBS for 24 h at 4 °C, and then transferred to a 30% (*m*/*v*) sucrose/PBS solution containing 0.02% (*m*/*v*) sodium azide for cryoprotection. Brain samples were embedded in O.C.T. compound (Sakura, Torrance, CA, USA) and frozen in isopentane cooled by liquid nitrogen. Frozen blocks were serially sectioned at 30 μm thickness in the coronal plane using a freezing microtome (Leica, Wetzlar, Germany). Brain sections were kept free-floating in PBS with 0.02% sodium azide at 4 °C. For dual immunolabelling experiments involving detection of intranuclear inclusions, brain tissue was drop-fixed overnight at 4 °C in 4% PFA/PBS, cryoprotected in 30% sucrose/PBS with 0.02% sodium azide, and embedded in O.C.T. compound before freezing in liquid nitrogen-cooled isopentane. The frozen tissue blocks were sectioned to seven μm thickness, mounted on poly-L-lysine coated slides (Roth), air-dried, and stored at 4 °C.

The sections underwent heat-induced antigen retrieval using 0.01M sodium citrate (pH = 6.0), heated in a microwave if mounted on slides or in a 95 °C hot air oven if free-floating. For inclusion labelling experiments, an additional proteolytic antigen retrieval step with proteinase K (5 μg/mL) was performed. The sections were first blocked for endogenous biotin using an avidin/biotin blocking kit (Vector, Newark, CA, USA) and then for mouse Ig when using mouse primary antibodies or with serum when primary antibodies from non-mouse species were used. The sections were incubated overnight at 4 °C with primary antibodies, followed by biotinylated secondary antibody incubation (Vector). Antigen–antibody complexes were then fluorescently labelled with either Cyanine 3 or Cyanine 5-conjugated streptavidin (Invitrogen, Carlsbad, CA, USA). Dual immunolabelling experiments were performed sequentially for each primary antibody. The sections were counterstained with DAPI and coverslipped with ImmuMount (Shandon, Runcorn, UK). The primary antibodies used were rabbit anti-tyrosine hydroxylase (1:100 *v/v*, sc-25269, Santa Cruz, CA, USA), rabbit anti-dopamine transporter (1:250, ab184451, Abcam, Cambridge, MA, USA), goat anti-green fluorescent protein (1:100, ab6673, Abcam), and mouse anti-FMRpolyG-8FM (1:200, a kind gift from N. Charlet-Berguerand), which was previously described by Buijsen and colleagues [46].

### 4.4. Imaging and Cell Count Analysis

Fluorescent photomicrographs of striatum and substantia nigra were captured at either at 100× or 400× magnification with a two μm z-step, using an epifluorescence microscope (Leica), and analyzed in Fiji open-source software version 1.54f. For signal intensity quantifications, 100× photomicrographs of the striatum and substantia nigra regions of interest were visually traced in Fiji and the software’s raw intensity and area measurement functions were used to report relative intensity densities normalized to the traced area. DAT stainings were quantified from 12 to 13 striatal sections per brain, while the TH signal quantifications involved five substantia nigra sections from each mouse. Values from individual sections were averaged to obtain a single measurement per mouse. For cell counts of DAPI and TH-positive cells in the substantia nigra, 400× photomicrographs were processed using a custom cell counter script developed for Fiji. The script partitioned traced regions of interest from tiled whole photomicrographs into 30 μm by 30 μm partial images, recorded the area of the traced region and displayed the partial images randomly. A researcher, blind to the experimental groups visually determined all DAPI-labelled and TH-immunoreactive cells contained in each partial image. With input from the researcher, the script recorded cell counts for every analyzed image and the number of counts per area within the traced region is reported.

### 4.5. Statistical Analysis

Statistical analyses were performed using the Prism statistical package (GraphPad Prism version 9.5.0). Data distribution was assessed using the Shapiro–Wilk normality test. For comparisons between groups with normally distributed data, an unpaired Student’s *t* test was applied. For data not meeting normality, the Mann–Whitney U test was used. When comparing two factors with repeated measures for one of the factors, a two-way repeated-measures ANOVA was conducted. A *p* value of less than 0.05 was considered statistically significant for all analyses with “*”, “**”, and “ns” indicating *p* < 0.05, *p* < 0.01, and *p* > 0.05, respectively.

## Data Availability

The data that support the findings of this study are available from the corresponding author, O.S., upon reasonable request.

## Supplementary Materials

The supporting information can be downloaded at https://www.mdpi.com/article/10.3390/ijms26041511/s1.
