# Supplementary material for: Nigrostriatal Degeneration Underpins Sensorimotor Dysfunction in an Inducible Mouse Model of Fragile X-Associated Tremor/Ataxia Syndrome (FXTAS)
Source: Int J Mol Sci. 2025 Feb 11;26(4):1511. doi: 10.3390/ijms26041511 (PMC11855849; doi:10.3390/ijms26041511)

**SUPPLEMENTARY FIGURES**

**SUPPLEMENTARY FIGURE LEGENDS**

**Supplementary Figure S1. Control Experiments: CGG-Size & Baseline Acoustic Startle Response**

**A.** Prepulse inhibition levels in P11CGG mice induced with doxycycline for 24 weeks were unaffected compared to non-induced control mice. **B.** Acoustic startle responses of P90CGG mice at sound pressure levels 80-110 dB were not affected by 12-week dox induction. **C.** Acoustic startle responses of P90CGG mice were unaffected by 24-week dox induction. **D.** No differences were observed at acoustic startle responses of the 24-week dox induced P11CGG mice as compared to non-induced controls. Data presented as mean ±S.E.M. ns: $p>0.05$.

**Supplementary Figure S2. Dopaminergic Cells in the SNpc express the 90CGG Repeat Tract**

**A.** Representative photomicrograph from dual immunolabelling experiments of coronal sections of the substantia nigra pars compacta (SNpc) of 24 weeks dox-induced P90CGG mice showing the overlap of TH (red) and GFP (green) signals. Scale bar indicates 200 μm. **B.** Close up caption from A (white inset), isolating the GFP signal. **C.** Close up caption from A (white inset), isolating the TH signal. **D.** Merged image of the captions from B and C, showcasing the overlap of GFP and TH signals (orange). Scale bar indicates 20 μm.

**Supplementary Figure S1.**

**Control Experiments: CGG-Size & Baseline Acoustic Startle Response**

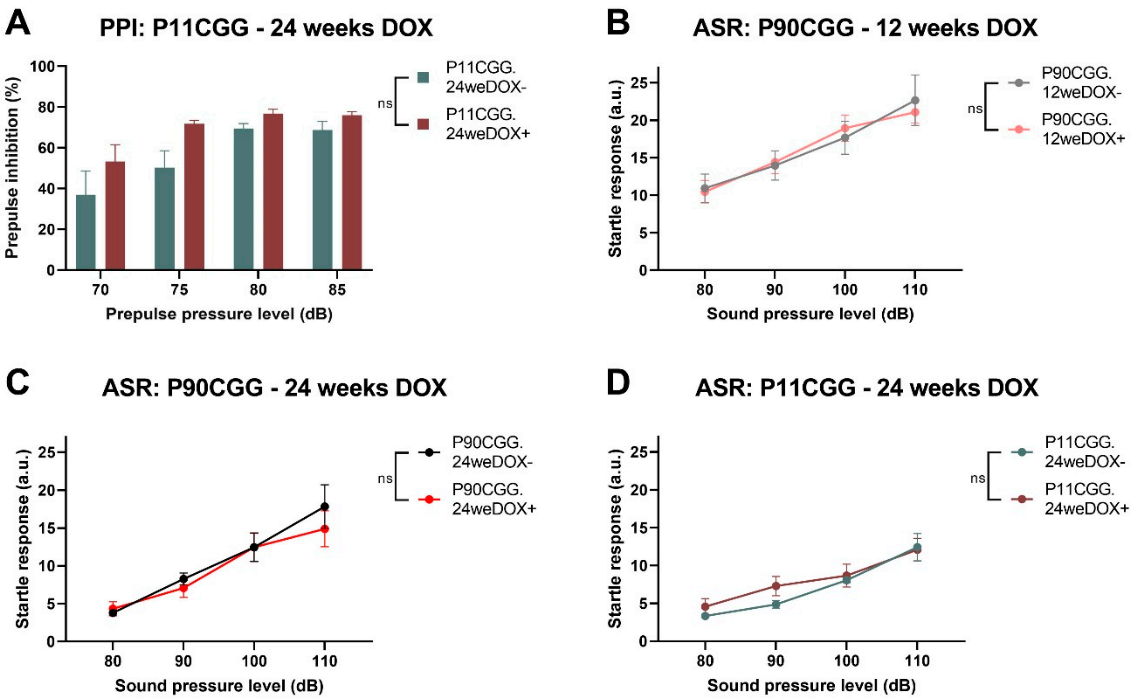

Supplementary Figure S2.

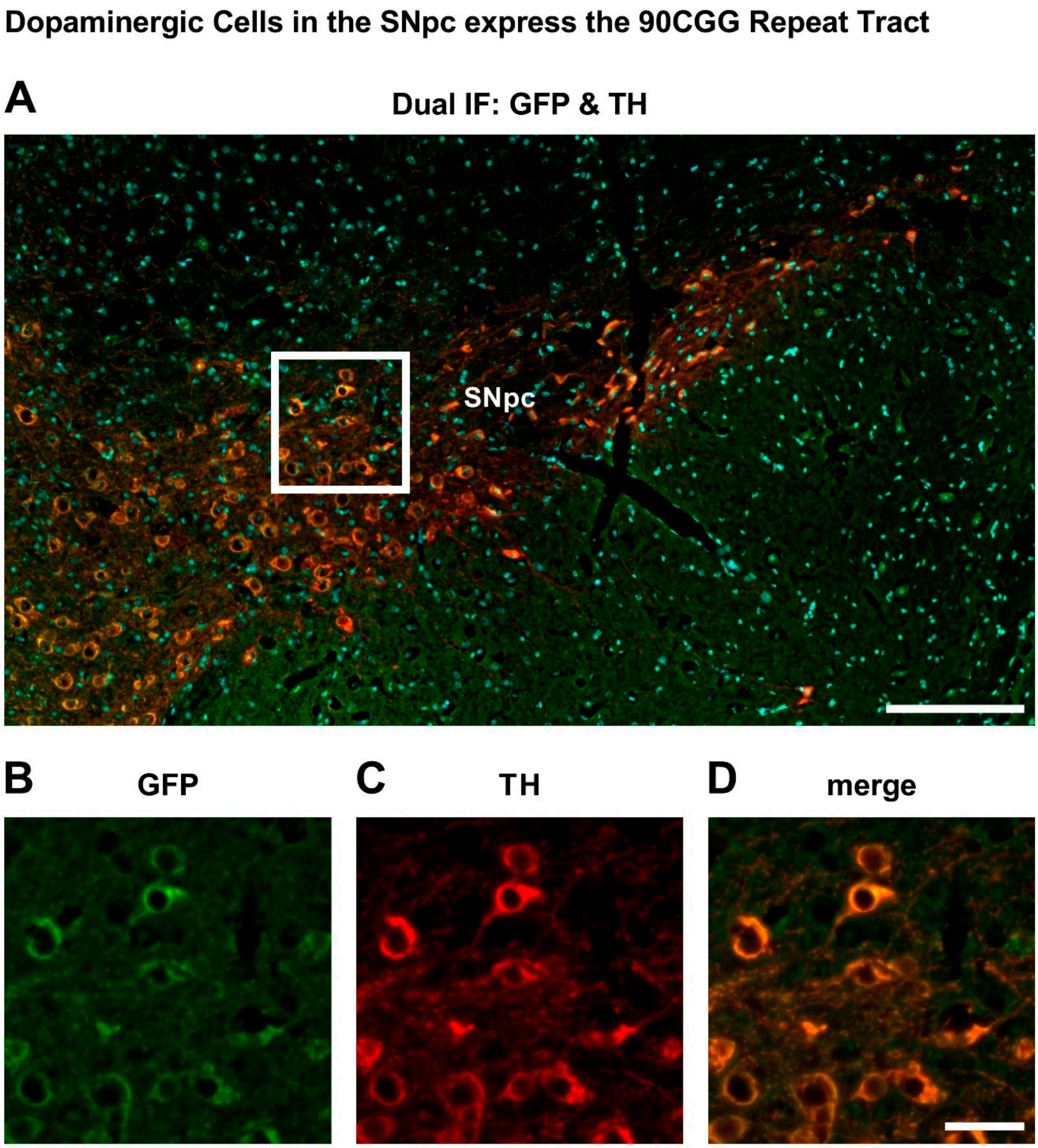

Supplement: Supplementary file 1 [file ijms-26-01511-s001.zip › ijms-3430993-supplementary.pdf]
